# Supplementary material for: PyOIF: Computational tool for modelling of multi-cell flows in complex geometries
Source: PLoS Comput Biol. 2020 Oct 19;16(10):e1008249. doi: 10.1371/journal.pcbi.1008249 (PMC7595628; doi:10.1371/journal.pcbi.1008249)
Supplement: Attachment — Submitted filename: response_to_reviewers.pdf [file pcbi.1008249.s003.pdf]

Point-to-point response to reviewers' comments

Manuscript:
PyOIF: Computational tool for modelling
of multi-cell flows in complex geometries

We would like to thank both reviewers for their comments. Both reviews pointed out some issue that we have not thought of before. The resulting changes certainly increased the quality of the paper. We incorporated most of the comments and in the following text we give the overview of the changes in the article.

Review 1

Comment: *The model calibration and validation is purely based on previous published papers and thus the section could be largely shorten. The general workflow section is based on the user guide documentation already available online with the source codes, while the design principles sound more a compilation of the advantages of the code provided with technical justification and/or evidence.*

Response: We agree that the user guide gives many details about the software implementation. However, the user guide mostly gives specific practical details and not the general picture. For researchers that need to pick a software to model their bio-physical problems, the user guide is too specific. The aim of this article is to give a brief overview of the software capabilities so that a researcher can make an educated decision regarding their software needs. With this in mind, the section 'Design principles and General workflow and implementation' was left unchanged and we think it is important to the readers to have such a summary in one place. We have significantly shortened the section 'Model calibration and validation', as suggested.

Comment: *In section 3, results, line 398, page 11, the authors explicitly state that those results 'have been thoroughly described elsewhere'. While they do not add the corresponding references, my question is thus as followed: what is the benefit for the community to have a paper repeating results that have already been published? For example, the case of section 3.1 has been published in ref[34]. Section 3.3 states that the authors 'are able to track more detailed information' (line 605, page 17), however, none of the listed information is actually presented in the paper. Moreover, only a video is provided and no quantitative data presented nor any validation for this case.*

Response: We think that summarizing several important findings from other papers that are relevant to description of the model, is not "repeating the results". Our primary goal was to showcase various types of questions that can be investigated using this software. We agree though that such summary may be shorter and we reduced the text accordingly. We distilled the crucial information from the sections 3.1 and 3.3 and put it into the section 3.2 'Cell flow in a bifurcation'. The numbering 3.2 thus became obsolete and was removed. This section was then extended with a time complexity study (motivated by the second review) and with several novel quantitative insights into cells flowing in bifurcations: evolution of cell's surface, diameter and rotation angle during the cell's passage through the bifurcation and time-evolution of local hematocrit values in different parts of the channel with respect to the initial conditions.

Comment: *From the video, it looks like the cells are spherical and do not have a discocyte shape for the most common shape of a healthy RBC. Can the authors explain if the code handles discocyte and other RBC shapes?*

Response: One of the three videos in the original supplementary material depicts the flow of spherical cells (rare cells, which are more rigid) and not the red blood cells. The other two videos showed discocyte-shaped red blood cells, but it was our intention to show that other closed 3D shapes can be used. However, we removed section 3.3 and so we removed this particular video as well.

To specifically answer the question about the possibility of modelling discocyte shapes: Yes, indeed. In the original submission, we provided the details in section 2 'Design and implementation', subsection 'Input-output'. Further, the simulation of RBC flow in bifurcating channel described in section 3 is an example that showcases thes cells. The shape of the cells is defined by two files: list of nodes with their coordinates, and list of surface triangles defined by triplets of nodes. Therefore the shape of the cells can also be adjusted by user, if needed.

Review 2

Comment: *The authors mention a couple of software packages, which may have similar/comparable possibilities. I think it would be useful to discuss similarities/differences advantages/disadvantages in more detail. Why should I choose the proposed software over another one? Maybe some sort of a summarizing table would be useful.*

Response: In the original submission, we provide a few paragraphs spanning one page (Section 1 Introduction, subsection Similar methods and tools) containing comparison of our model vs other models, as well as our software vs other softwares. We also state advantages and disadvantages of the models and softwares.

Providing an additional table summarizing this text does not seem necessary, especially in view of editor's and the first reviewer's comments about shortening the manuscript.

Comment: *Clearly, sophisticated models are complex and have a number of parameters. Therefore, it is probably impossible to use them as black boxes and some level of understanding is needed. The authors mention several times that certain parameters were calibrated in previous works. Does a user need to understand all these previously performed tests, in order to have a good idea of possible pitfalls? Would the software give some suggestions or warnings?*

Response: There are some model parameters that can in principle be used as a black box and other that need to be defined with more background knowledge. For example, the user does not have to fully understand the calibration of the five elastic moduli, however, the cell-wall, or cell-cell interaction coefficients need to be set with consideration to the fluid flow and elasticity of the cells. Currently, there is an internal discussion in our group to decide whether our software module should contain a command that would create an "out-of-the-box" red blood cell with predefined parameters that would not require any deeper knowledge. However, we are still not convinced about the net positive effect of such feature and whether it would be a useful and desirable addition. With regard to warnings, we try to follow the Espresso philosophy of having methods that either succeed or fail.

Comment: *It is difficult to judge software performance. Therefore, I would like to ask the authors to mention explicitly the performance numbers for different tests. How long each simulation takes? How well it is paralleled (speed-up, strong and weak scaling)? What is the most expensive part LBM or cell modeling? I think it would be useful to give running times for different examples to have an idea of the performance.*

Response: We added a subsection in Section 3 that discusses the time complexity of the bifurcation simulation.

Comment: *Are you planning collective software development, so that users can contribute to this? How can you enable this efficiently?*

Response: Our software is open-source, available on github and we are open to cooperation with other contributors who would like to add new features to the PyOIF module. The questions related to PyOIF module posted to the Espresso mailinglists (user or developer) are typically answered by PyOIF developers who are actively using the module. There has also been development of new features per users' requests. We added some remarks about this topic into section 4 'Availability and future directions'.
